# Desiccated Suprarenal Capsule in Acute Coryza1Read before the Surgical Section of the Buffalo Academy of Medicine, December 5, 1899.

**Published:** 1900-03

**Authors:** Frederick H. Millener

**Affiliations:** Buffalo, N. Y.


### DESICCATED SUPRARENAL CAPSULE IN ACUTE CORYZA.[1]

By FREDERICK H. MILLENER, M. D., Buffalo, N. Y.

I WISH to beg the indulgence of the Academy for presenting a subject which is apparently so trivial as the relief of an acute coryza. An eminent professor, I believe it was Professor Hare, of Philadelphia, once remarked, that the reason our homeopathic brethren are so uniformily successful in their practice from both a worldly and medical point of view, is not because their theory and practice is proper and scientifically correct, but because of their attention to details, and it is certainly not that they cure disease, but rather that they alleviate the painful symptoms and allow nature to effect the cure, for as soon as the pain is gone the patient is patient indeed.

During the last year or two much work has been performed for the determination of the function of the suprarenal capsules, and of their possible use in therapeutics. It is not yet settled whether or not they contain a true active principle, much less what that active principle is. They certainly contain some toxic substance. It is demonstrated from the action possessed by the suprarenal capsules, or their extract, that they contain an active principle which is the most powerful substance known as a direct or indirect stimulant of the vaso-motor system, resulting in a decided rise of the blood pressure. This great increase in arterial pressure, after the ingestion of this gland, or its

1 Read before the Surgical Section of the Buffalo Academy of Medicine, December 5, 1899.

extracts, is due to the extraordinary power they possess over the muscular fibers in the walls of the arterioles. One tenth of a grain of the desiccated gland causes a maximal effect upon the heart and arteries of a twenty pound dog. From this the conclusion is drawn, that its principle is capable of occasioning a well-marked physiological effect, in the dose of one eight-hundredth of a grain, in an adult man. In the *Wiener Medizinsche Blatter*, November 11, 1897, Velich points to the local anemia in mucous membranes occasioned by the local application of the suprarenal extract. He described the action of the extract on the unbroken skin and also arrived at the conclusion that this preparation, when applied to the broken or unbroken skin, produces a local anemia. He has observed that the normal florid color of the skin vanishes, and draws attention to its effects if applied to portions of the epidermis affected with eczema.

It is extremely interesting to note that when the drug is applied to epidermis reddened by eczema or scalding, its action is always confined to the site of its application. The aqueous extract, according to Bates, is a powerful astringent and hemostatic. Internally, it increases the action of the heart, but considerable quantities may be ingested without apparent injury. Its use hypodermatically requires careful watching; locally applied, the drug is never followed by any constitutional symptoms or manifestations. In an instance referred to in the authority just mentioned, ten grains occasioned alarming effects—the face became livid and there were throbbing pains in the head and chest; the pulse was weak, but consciousness remained clear and the patient soon recovered.

Bates further states, "when it is instilled into the eye the conjunctiva of the globe and lids is whitened in a few moments." It has been noticed that the same effect takes place in the nose and naso-pharynx and none of the usual drugs, including cocaine, can produce such an astringent effect. It was therefore applied to a congested condition of the nasal mucous membrane such as is present in an acute coryza. The aqueous extract was prepared by dissolving twenty grains of the dried extract in half an ounce of water and filtering through cotton. The drug should be applied by first cleansing the nose and naso-pharynx with an alkaline wash made up of sodium salicylate, sodium bicarbonate and boracic acid. The aqueous extract of suprarenal capsule is applied to the nasal membrane on pledgets of cotton, and posteriorly to the naso-pharynx by means of an atomiser.

In order to get the best results the extract should be made up fresh as it will not keep in a warm room, and if it is dissolved in a saturated solution of boracic acid it does not work as satisfactorily. The patient is thus relieved of a very distressing condition for two or three hours, or until such time as the patient may use a mercurial purgative or otherwise get rid of this condition. I merely wish to state that extract of suprarenal capsule may take the place of that dangerous drug cocaine with great success for relief of congested mucous membrane. The exract may also be employed in some of the more or less bloody operations on the nose. The extract prevents hemorrhage because of its property of contracting the small arteries and arterioles. After hemorrhage begins the extract is not efficient.

It is possible to perform almost any operation in the nose or on the nasal membrane almost bloodlessly, providing you insert a pledget of cotton wool soaked with 5 per cent. solution of cocaine into each nostril, leaving them in *situ* for five minutes. On withdrawing these, similar pledgets soaked in solution containing five grains of suprarenal gland to the ounce are introduced and left there for the same length of time. The extract is not an objectionable hemostatic. It does not form clots like iron or irritate as does peroxide of hydrogen.

5 North Pearl Street.

